# Breastfeeding and feminism: A focus on reproductive health, rights and justice

**DOI:** 10.1186/1746-4358-3-8

**Published:** 2008-08-04

**Authors:** Miriam H Labbok, Paige Hall Smith, Emily C Taylor

**Affiliations:** 1 Center for Infant and Young Child Feeding and Care, Department of Maternal and Child Health, University of North Carolina School of Public Health, Chapel Hill, North Carolina, USA; 2 Center for Women's Health and Wellness, Department of Public Health Education, School of Health and Human Performance, University of North Carolina at Greensboro, Greensboro, North Carolina, USA

## Abstract

The annual Breastfeeding and Feminism Symposia aim to reposition breastfeeding as a valued part of women's (re)productive lives and rights. The symposia are designed to raise the profile of breastfeeding within the women's advocacy and feminist studies' communities, and to increase recognition among breastfeeding supporters that breastfeeding promotion could receive more socio-political support by partnering with those concerned with women's reproductive health, rights and justice, women's economic advancement, and the elimination of social, economic and health inequities. The third symposium (2007) sought to build dialogue and increase communications between and among these diverse communities. The nine articles presented in this thematic series were selected by the journal editors, and represent the core discussions at the symposium. This editorial presents the areas of synergy and strategies for action that emerged from the discussions. These strategies and this thematic issue are intended to reassert the momentum that evolved among participants, and to stimulate involvement among individuals and organizations not in attendance in promoting breastfeeding as a women's reproductive health, rights and justice concern.

## Introduction

The annual Breastfeeding and Feminism Symposia were initiated with the aim to reposition breastfeeding as a valued part of women's reproductive rights and lives. Each year, the effort is made to raise the profile of breastfeeding within women's advocacy and feminist studies' communities. These symposia are also designed to increase recognition among breastfeeding supporters that breastfeeding promotion could receive more socio-political support by partnering with those concerned with women's reproductive health, rights and justice, women's economic advancement, and the elimination of social, economic and health inequities.

The Third Annual Breastfeeding and Feminism Symposium, September 24 and 25, 2007, was held in Chapel Hill, North Carolina and was co-hosted by the Center for Women's Health and Wellness, University of North Carolina, Greensboro (founding organization), and Center for Infant and Young Child Feeding and Care, School of Public Health, University of North Carolina at Chapel Hill. This event included both presentations and working groups to build dialogue and increase communications between and among feminists, policymakers, researchers, breastfeeding advocates and practitioners to help promote breastfeeding as a woman's reproductive health, rights and justice concern.

Although research on breastfeeding has established that it is a maternal and child health imperative, yielding optimal short and long term health outcomes for both mother and child, breastfeeding is not fully recognized as a feminist, women's rights or women's reproductive health concern. Most second wave feminist scholarship and activism has presented breastfeeding as an "option" or a "choice" that is generally presented as not very different from formula feeding. A limited number within the feminist community has recognized breastfeeding as a women's health issue or a reproductive right. In fact, global support for women's rights generally ignores the rights and importance associated with all of women's roles as mothers, opting instead to concentrate primarily on other important issues such as employment and reproductive freedom.

The 2007 symposium on Breastfeeding and Feminism focused on reproductive health, rights and justice, and was intended to stimulate consideration and identification of areas of mutual interest across diverse groups, including feminists, health workers, public health planners, community members, mothers and breastfeeding specialists. It was also designed to serve as a catalyst to strengthen coalitions and synergy to further breastfeeding as a reproductive right, and to create the first steps in action planning.

The presentations and working group discussions were based on the following principles:

• Breastfeeding is a social *and *biological process wherein women must have the right of self-determination;

• Breastfeeding is a maternal and child health imperative and reproductive right;

• It is important to re-orient the paradigm from the current view that breastfeeding is a "lifestyle choice," to a paradigm that views breastfeeding as a reproductive health, rights and social justice issue so as to ensure the social, economic and political conditions necessary to promote success;

• Women's decisions to breastfeed should not result in the loss of their economic security or any rights or privileges to which they are otherwise entitled.

The nine articles presented in this thematic series were selected by the journal editors from among the proceedings of this symposium and workshop, and cover the major areas of discussion. This series of articles provides a basis to understand the momentum that evolved among participants, and are presented to stimulate involvement among individuals and organizations not in attendance. The full proceedings of the symposium and working sessions are available for citation on the websites of the co-hosts [1,2].

## Outcomes and actions: transdisciplinary identification of common goals and multi-disciplinary discussion of feasible actions

Two working group sessions were held, the first to explore areas of synergy and the second to begin the development of strategies for action. The first session grouped participants across disciplines, but with an interest in a) reproductive health, b) reproductive rights, or c) reproductive justice. During this session, groups were tasked with discovering areas of synergy between or among: a) breastfeeding, b) feminism, and c) reproductive health, rights or justice, in order to identify goals and areas of need.

Working group outcomes and other discussions at the conference indicate that participants recognize the following goals:

1) the economic, political and cultural connections between women's rights to have children, *and *their rights not to have children;

2) that women place importance and value on being able to mother in ways that are consistent with their own values, *and *on being creatively and productively engaged in the labor force; and

3) that the decision to breastfeed is not as yet a realistic "choice" for many women if not supported by policies and programs that provide *all *women, regardless of their socio-economic status, with education, opportunity, health system and socio-political support, and control over their bodies and lives.

Conference participants identified the need for political, health care and cultural changes that support women's full participation in society as reproductive and productive beings, increase the value placed on women's reproductive abilities and secure their rights across a reproductive continuum that includes a full range of family planning services, clinical abortion, pregnancy and birth treated as part of a health continuum and breastfeeding.

Four areas related to women's reproductive health emerged in this transdisciplinary discussion that could contribute toward these goals. These include the need to:

(1) create a mother and breastfeeding friendly health care system that re-enforces breastfeeding as the normative approach to infant feeding;

(2) train all health workers to support reproduction as health, not illness;

(3) secure adequate regulation of infant formula marketing to health workers and to the public, and of pharmaceutical labeling as concerns the mother-child dyad; and

(4) promote a culture and civil society that value women as whole beings.

Four areas emerged from discussion that relates to women's reproductive rights, including the need to:

(1) recognize the importance and value of women's rights across the reproductive continuum;

(2) recognize the importance and value of women's reproductive and productive roles;

(3) create mother-friendly workplaces, including paid maternity leave with guarantee of return; and

(4) engage the US as a global partner in human rights efforts.

The two identified areas related to women's reproductive justice were the need to: (1) secure economic justice for women; and (2) secure racial and ethnic equality.

These areas of need that emerged as a result of transdisciplinary discussion, rather than sector-based discussion, serve as a starting point for formation of partnerships and coalitions for action to work together toward common goals.

Participants self-selected by professional discipline into one of three groupings for the second set of working groups: a) academic/researchers, b) policy/legal expertise, or c) practitioners/service providers. These groups started with a brief review of the outcomes of the earlier discussions and went on to identify short and long term strategies for action.

The working group participants identified numerous strategic actions intended to create change at different levels of the socio-ecological model. (See Labbok article in this thematic series for a discussion of the socio-ecological model).

• **Individual/family level: **Support recognition of the cost-benefits of mother/child attentive reproductive health support and breastfeeding for the family, as well as for the mother and child.

• **Community/cultural level: **The suggested actions include: (1) engage in broad-based breastfeeding education (targeting the public, students, clinicians, employers, unions and policymakers); (2) raise public awareness of infant formula companies' marketing strategies and influence on all sectors; (3) increasing all women's access to economic resources and opportunities to achieve reproductive goals based on unbiased information.

• **Organizational level: **Overall, everyone interested in reproductive health, rights or justice must work together to secure the respect, rights and opportunities for women from "the birthplace to the workplace". Such actions related to health care organizations include the need to: (1) support the Baby Friendly Hospital Initiative; (2) improve women's access to midwifery care and other sources of woman-centered, holistic, integrative models of care. Necessary actions related to workplaces include: (1) secure workplace support including: paid maternity leave; onsite child care; flexible hours and home work; mothers' rooms; and separate paid infant feeding breaks in addition to other breaks; (2) dissemination of available documents related to costs and benefits of breastfeeding, and the costs and risks of alternate feeding, to appropriate policymakers, employers, consumers (e.g. the dissemination of the *Business Case for Breastfeeding *by the US Department of Health and Human Services). Actions suggested for universities and other research settings include: (1) reducing academic and professional "silos" in favor of transdisciplinary identification of problems and gaps, multi-disciplinary research to address these issues, evidence creation through translational and applied studies, knowledge dissemination, and professional practice; (2) examining and increasing understanding of the "lived realities" of diverse populations of women as related to mothering, breastfeeding and employment; (3) developing a scientific working group to clarify defensible, ethical and safe methods for research on the effects of medications on lactation, and to further understand the risks of not breastfeeding; and (4) supporting family practice within the health fields to increase attention to the reality of the mother/child dyad.

• **Policy level: **There is a need to seek passage of currently proposed legislation in the U.S. such as the Breastfeeding Promotion Act, the Global Childhood Survival Act and the Emergency Contraceptive Education Act, and to work toward the development and passage of legislation to reduce aggressive and misleading infant formula industry marketing, to regulate and support growth of donor milk banking, to create equitable health insurance that is not employment-based, and to expand the Family and Medical Leave Act to address all maternal/child needs. To be part of the global community, work toward the US ratification of United Nations Convention on the Rights of the Child and the Convention to Eliminate Discrimination Against Women is needed.

Table 1 summarizes the areas of synergy and strategies for action that emerged from these working sessions.

**Table 1:** Areas of synergy and strategies for action to improve women's reproductive health, rights and justice

***Seeking Synergy through Transdisciplinary Discussion of Gaps and Needs: *Overarching themes that emerged, connecting breastfeeding to women's reproductive health, rights and justice**			
• There are the economic, political and cultural connections between women's rights to have children, *and *their rights not to have children			
• Women place importance and value on being able to mother in ways that are consistent with their own values, *and *on being creatively and productively engaged in the labor force			
• The decision to breastfeed is not a "real choice" for many women if not supported by policies and programs that provide *all *women, regardless of their social position, with education, opportunity, and control over their bodies and lives.			

***Goals and Areas of Need***			

**Reproductive Health**	**Reproductive Rights**		**Reproductive Justice**
• Create a mother-friendly health care system	• Recognize the importance and value of women's reproductive and productive roles.		• Secure economic justice for women
• Value women as whole beings	• Create a mother-friendly workplace		• Secure racial and ethnic equality
	• Secure better governmental oversight over pharmaceutical labeling and infant formula		
	• Engage the US as a global partner in human rights efforts		

***Strategies for Action across the Socio-ecological Levels***			

***Individual/Family***	***Socio-Cultural***	***Organizational, by Sector***	***Policy***

• Support recognition by individuals and by families of the costs and benefits of mother/child attentive support throughout the reproductive health continuum.	• Provide broad based education on benefits and practice of breastfeeding for:	Health care system:	Initiate and approve legislation to enable breastfeeding, including:
• Ensure that families understand the potential impact of less than optimal infant feeding on the health and welfare of mothers, children and families.	• Public	• Baby Friendly Hospital Initiative	• Breastfeeding promotion and incentives
	• Clinicians	• Access to midwives and holistic care	• Family planning access
	• Employers	Worksites:	• Donor milk banking
	• Unions	• Maternity leave	• Health insurance coverage for lactation services
	• Policymakers	• Onsite child care	• Paid maternity, family and medical leave
	• Raise public awareness of infant formula companies' practices	• Support for breastfeeding and pumping	Encourage US Government ratification of UN Conventions:
	• Increase women's access to resources and opportunities	• Flexible hours and home work	• Convention on the Rights of the Child
	• Continue Breastfeeding and Feminism Symposia	• Part-time work with benefits	• Convention for the Elimination of Discrimination against Women
		Universities/Research Centers:	
		• Reduce academic "silos"	
		• Collaborative partners	
		• Evidence and knowledge based dissemination	
		• Research on "lived realities" of diverse populations	
		• Scientific working group on ethical and safe methods for researching impact of medications on lactation	

## Tracking progress

In the months that followed the Breastfeeding and Feminism Symposium, organizers continued to hear from enthusiastic participants. Some emails came simply in the form of high praise for the event, and expressions of high hopes for future partnerships, while others were searching for additional information to bring back to their organizations and communities. Many reported on progress made toward the goals and action steps identified at the meeting. Still others reported on progress made toward new objectives inspired by the ideas exchanged and the group work during the symposium.

The following is a brief summary of the reports received by conference organizers:

### Actions taken in support of the individual and family

• Local advocates and professionals working in family planning, pregnancy, childbirth, and breastfeeding gathered in Chapel Hill to celebrate the common location of their individual "issues" on the reproductive continuum. A listserv was created for sharing information between groups, in hopes of "breaking down silos."

• Many Lactation Consultants and nurses expressed a heightened awareness of the barriers to breastfeeding, leading them to be more supportive of mothers trying to overcome obstacles to breastfeeding, predominantly by counseling on possible barriers that they may face, and by supporting the actions needed and the strength and self-efficacy to overcome them.

### Actions taken in support of the community/societal/cultural level change

#### Academic

• Four participants created and presented, "Disturbing the 'Public': Risks, Rights, Breastfeeding, and Feminism" at the Southeastern Women's Studies Association at University of North Carolina, Charlotte.

#### Health care

• Two hospitals in North Carolina report being "close to BFHI certification."

• The North Carolina Breastfeeding Coalition launched a campaign to reduce infant formula companies' advertising through maternity centers. As of July 1, 2008, five hospitals will be acknowledged with "Golden Bow Awards" for eliminating all such advertising.

• The Durham County Health Department has enhanced its programs to include breastfeeding goal setting and additional support for breastfeeding pairs with the objective of increasing breastfeeding rates among minority and low-income clients.

#### Workplace

• Two breakfast table discussions about employer recognition awards (ERAs) were held at the National Conference of State Breastfeeding Coalitions in January 2008. This was seen as an opportunity to find out what State Breastfeeding Coalitions are doing in this area, with the hope of developing replicable models for use in other states.

• Participants participated in evaluating "The Business Case for Breastfeeding," a toolkit developed by the Maternal and Child Health Bureau for training breastfeeding advocates to reach out to employers in support of women in the workplace.

### Actions taken in support of governmental/policy change

• One group provided ongoing consultation to a state task force to ensure inclusion of breastfeeding in legislated Safe Sleep messaging in North Carolina.

• Many individuals are engaging in grass roots advocacy for Carolyn Maloney's Family and Medical Leave Act.

• The New Jersey Breastfeeding Task Force is participating in the Time to Care Coalition, a broad-based coalition campaigning for The Family and Medical Leave Act.

• The New Jersey Breastfeeding Task Force is partnering with the Rutgers Center for Women and Work, New Jersey National Organization for Women (NOW) and Mothering-NOW, and mothers' organizations such as "Mothers and More" to advance The Family and Medical Leave Act.

• Individuals and organizations in North Carolina, Pennsylvania, and Vermont report working on creating and advancing state-level legislation to support, promote and protect breastfeeding.

• For the 2008 meeting of the UN's Commission on the Status of Women (CSW), UN Breastfeeding Action Team (UNBAT), the UN breastfeeding advocacy team made up of representatives from La Leche League International, International Lactation Consultants Association, Academy of Breastfeeding Medicine and World Alliance for Breastfeeding Action, prepared a statement on the theme for 2008, "Financing for gender equality and the empowerment of women". The purpose of the statement was to demonstrate, in a one-page format, the value of breastfeeding as food, health care and childcare. It was entitled, "The Breastfeeding Budget," and was based on the publications of Australian economist Julie Smith and her colleagues. In order to increase attention to the statement, UNBAT placed an advertisement about it in the CSW program book.

This list of actions is quite impressive, both in its content, and in the fact that it reflects a commitment to synergy across the reproductive health continuum and among the individuals and groups who came together at the Breastfeeding and Feminism Symposium. While Symposium objectives were achieved, there is still much work to be done. Plans are underway for the Fourth Breastfeeding and Feminism Symposium to be held in March 2009 at University of North Carolina, Greensboro, where our theme will be "sustaining breastfeeding from the birthplace to the workplace in support of healthy mothers, healthy babies and healthy environments."

## Competing interests

The authors declare that they have no competing interests.

## Authors' contributions

All three authors shared in conceptualizing the themes and working group agendae for the symposium. PHS was responsible for writing the abstract and introduction, and created Table 1. ECT organized and reported on working group outcomes and progress reports from symposium participants. MHL edited the near-complete draft for clarity and uniformity of style.
